# A Case of Recovery after Extensive Contusion and Laceration of the Perinæum and Urethra

**Published:** 1884-05

**Authors:** W. H. Washburne

**Affiliations:** Florence, Wis.


### ARTICLE II.

### A CASE OF RECOVERY AFTER EXTENSIVE CONTUSION AND LACERATION OF THE PERINÆUM AND URETHRA. By W. H. WASHBURNE, M.D., Florence, Wis.

J. M., a healthy man aged twenty-five years, a member of the voluntary fire department of Florence, while in the discharge of the duties of a fireman at a fire on the evening of Nov. 26, 1883, entered the second story of the burning building. The floor gave way under him, and he was precipitated to the ground floor, falling astride a floor joist, projecting from which was a splinter of wood.

He walked to his boarding house, a distance of a quarter of a mile, with his clothing wet through and frozen. I saw him immediately, found him suffering from great shock; small, weak pulse; and shivering with the cold.

Upon examination, I found the end of a charred piece of wood projecting from a wound in the right ischio-rectal fossa. This was immediately withdrawn, and the usual means adopted to encourage reaction from the shock.

The splinter measured 5½ inches in length, 1½ inches in width, and 1 inch in thickness at the thickest point. The splinter was driven into the soft parts 3¾ inches. The wound was cleansed and dressed with carbolized olive oil (ʒi to ℥vi), and morphia administered at intervals during the night.

On the following morning the patient complained that he could not micturate, and the bladder was considerably distended. I attempted to introduce a catheter, but did not succeed, observing, as the instrument was withdrawn, that the eyelet was filled with charcoal and blood. This fact confirmed my fears that the urethra had been wounded.

At 2 o'clock P. M. the patient was anæsthetized, and with the assistance of Drs. C. A. Fortier and R. W. Odell, a thorough exploration of the wound was made. It was ascertained that the splinter had made an opening about two inches long, commencing an inch to the front and right of the anus, and extending backwards and to the right, that it passed obliquely upwards and forwards, tearing away a part of the bulbous and the whole of the membranous portion of the urethra, and passing between the symphysis pubis and the bladder, so that at least two inches of the urethral canal was utterly annihilated.

With a great deal of difficulty the vesical end of the urethra was found, and a No. 8 silver catheter introduced into the bladder and tied there. The patient passed through the catheter a large quantity of urine. No evidence of any infiltration of urine.

Patient passed a comparatively comfortable night, and Nov. 28, 9 A. M., pulse 84, temperature 100½° F. No hypogastric tenderness. Considerable ecchymosis appeared in the vicinity of the external wound. Prescribed quinine and morphia.

On the fourth day after the receipt of the injury his bowels moved, as the result of the administration of saline cathartics.

Previous attempts had been made to move his bowels by means of enemata, but in consequence of the injury to the sphincter ani the fluid was not retained, and no movement followed.

For two days all the urine was voided through the catheter, when it began to escape through the wound.

Dec. 6. Catheter still in the bladder; it moves in and out for a distance of an inch and a half, with changes in the patient's position, and seems to cause no undue irritation. In view of the extreme difficulty encountered in the introduction of the catheter, it was deemed the lesser of two evils to allow the instrument to remain in position for some days longer, or until it shows some indication of producing serious irritation. At no time up to date has the patient exhibited a temperature above 102½° F., and his pulse has not been above 90.

Dec. 7. This morning at 3 o'clock patient had a slight chill, complained of great uneasiness and thirst, and at the time he was seen at 8.30 A. M. his pulse was 96 and temperature 104½° F. Ordered quinine to be increased to six grains, repeated every three hours, given in capsules. Previous to the occurrence of this chill the patient had taken a seidlitz powder, which had not yet operated. At 6 o'clock in the morning he took one ounce of Rochelle salt, which operated thoroughly at 8 o'clock, and again at 2 p. m. The evening temperature, 8 P. M., was 103⅕° F., pulse 84. Ordered quinine continued in the same doses as ordered in the morning. At 9.30 o'clock in the evening was summoned to the patient, with the intelligence that he was having another chill. On arrival found that his chill had been but slight, but that his temperature had increased ; he complained of intense pain in the head; pulse strong and 90 per minute. He complained of some abdominal pain, which he described as lancinating. Prescribed morph. sulph. gr. ⅓ and pot. brom. gr. 20, to be given every three hours. This procured him a good night's sleep, better than he has enjoyed since the receipt of the injury.

Dec. 8, 8.30 A. M., found patient feeling quite comfortable; no pain anywhere. No tenderness of the abdomen. Pulse soft, compressible, and beating 72 per minute, temperature 99⅘°. Advised the continuance of quinine in six grain doses every four hours for to-day ; no evidence of cinchonism.

Dec. 9, pulse 60 ; temperature 98° both morning and evening.

Dec. 10, pulse 60 ; temperature 98° both morning and evening.

To-day an effort was made to introduce a filiform bougie (made extempore from a piece of whalebone) through the catheter into the bladder, with a view of leaving it there as a guide over which to introduce a flexible catheter. The effort was unsuccessful, it being impossible to pass the filiform bougie through the eyelet of the catheter.

It was not thought best to remove the instrument to-day. The only irritation which it appears to have created is at the end of the penis, the glans being somewhat reddened, and the prepuce in the region of the frænum being red and tumefied. When the catheter is forced up by changes in the patient's decubitus, it is wiped clean and carefully oiled, with a view to lessening the amount of irritation. The patient is now enjoying a good appetite, indulging in beef steak and potatoes with great relish.

Dec. 12, pulse 60; temperature normal. The tumefaction about the prepuce and irritation of the glans have now almost entirely disappeared. The bowels were again moved to-day by means of saline cathartics. While at stool and straining, the patient passed a considerable quantity of urine through the penis outside the catheter, the water coming through pretty freely.

Dec. 14. Renewed efforts have been made to pass the filiform bougie through the catheter, but without success. The patient complains of considerable pain along the urethra, and any voluntary effort to expel his urine is attended with pain more or less severe. No more urine has come through the urethra. The amount voided by the wound is now constantly decreasing, by far the larger portion escaping through the catheter. The swelling and redness about the glans penis have now entirely disappeared.

Dec. 17. Patient has not passed any urine through the wound since 5 P. M., Dec. 15, the whole amount coming freely through the catheter. There is no evidence of any infiltration of urine among the perineal tissues. The appearance of the wound externally is all that could be desired. The original dressing of carbolized olive oil still maintained. Patient eats and sleeps well, but still complains of pain when he makes voluntary efforts at urination.

Dec. 21. Patient continues to improve daily, the urine all coming through the catheter freely, none through the wound. The patient is now able to urinate at will, the eyelet of the instrument being outside the bladder. To-day, and for the past two days, at least half of his urine has been voided through the urethra outside the catheter. He now complains of no pain on micturition except in the urethra, about an inch back from the meatus, at the point where ten days ago there was so much soreness and tumefaction of the prepuce. His appetite is excellent, and the bowels move every day without the aid of medicine; sleeps well.

Dec. 24. Yesterday removed the catheter, it being the twenty-sixth day since its introduction. It came away very readily, being blackened its entire length. Near the eyelets there was slight roughening of its surface, but not enough to create any serious irritation. The internal surface was heavily coated with calcareous deposit, so that before a great while its channel would have been entirely closed.

Immediately upon the removal of this catheter, an elastic No. 8 was inserted in its place, its introduction being effected with the utmost facility. This catheter was allowed to remain in place until this morning, when it was withdrawn, and the patient allowed to urinate naturally. The urine came in a good-sized stream and with good force, and the patient complained of no pain except near the meatus, where there appears to be an ulceration. It is now deemed best not to keep a catheter continuously in the bladder, but instead thereof to introduce a sound at intervals of six hours, and maintain in position five or ten minutes. The patient was permitted to get out of bed this morning for the first time and stand upon his feet. He affirmed that it gave him no pain anywhere.

Dec. 27. Patient was up and dressed on Christmas day, and has been up every day since, remaining up all day.

The VanBuren sound No. 8 is now used morning and evening, and held in place one hour each time. The urethra is considerably contracted at the meatus, about an inch from the meatus, and at the seat of the injury, so that the introduction of the sound is attended by considerable pain.

Jan. 10, 1884. The patient has been taught to use the sound himself, and has used it twice daily since Jan. 1. He is now attending to his own business, being proprietor of a livery stable, and suffers no inconvenience so far as his work is concerned, as the result of his extensive injury. The external wound is nearly healed, and his physical appearance gives no evidence of the fearful ordeal through which he has passed * * *

Before the catheter was withdrawn from his bladder, the man began having partial erections which were very painful, particularly at those points described as giving evidence of the narrowing of the urethra, the case presenting some of the features of chordee. Since the withdrawal of the catheter, erections have been accompanied with constantly decreasing pain, but they are not perfect. From the general direction of the wound, it is fair to infer that Cowper's glands were injured, if not entirely destroyed, and it is doubtful whether the prostate gland escaped injury. The testicles were not injured, nor were the spermatic cords involved.

April 1, 1884. The patient has continued in perfect health to this date, his erections have grown more and more perfect until the present time, so that his potency is fully established and his recovery complete.

The history of this case is remarkable : 1st, For the rapidity with which so extensive and dangerous an injury was repaired ; 2nd, For the length of time the catheter was maintained in position (26 days) without producing any serious irritation ; 3rd, That on the sixteenth day after the receipt of his injury, the processes of repair had so far advanced that he passed his urine through the natural channels, and after the nineteenth day no more urine escaped through the wound ; 4th, That at no time was there any indication of peritonitis or cystitis; 5th, That with the exception of one day, there was but little constitutional disturbance ; 6th, That when the external wound closed, there was no infiltration of urine ; 7th, That in forty days he was attending to his usual avocations.

